# Coexistence of chaotic attractor and unstable limit cycles in a 3D dynamical system

**DOI:** 10.12688/openreseurope.13590.1

**Published:** 2021-05-17

**Authors:** Dana Constantinescu, Gheorghe Tigan, Xiang Zhang

**Affiliations:** 1 Department of Applied Mathematics, University of Craiova, Craiova, 200585, Romania; 2 Department of Mathematics, Politehnica University of Timisoara, Timisoara, 300006, Romania; 3 School of Mathematical Sciences, Shanghai Jiao Tong University, Shanghai, 200240, China

**Keywords:** chaotic attractors, bifurcation, limit cycle, algebraic invariant surfaces, global dynamics

## Abstract

The coexistence of stable limit cycles and chaotic attractors has already been observed in some 3D dynamical systems. In this paper we show, using the T-system, that unstable limit cycles and chaotic attractors can also coexist. Moreover, by completing the characterization of the existence of invariant algebraic surfaces and their associated global dynamics, we give a better understanding on the disappearance of the strange attractor and the limit cycles of the studied system.

## Introduction

The Lorenz system is considered a benchmark for systems with complex (chaotic) dynamics. It was one of the first examples of 3D autonomous differential systems showing the existence of a strange attractor. The contrast between the simplicity of its equations and the complexity of its dynamical behavior attracted a lot of attention since its publication in 1963, but its thorough study was possible only when computational devices were developed enough to support long and laborious numerical simulations.

Many other systems with chaotic behavior and simple analytical form were obtained later, such as: the Rössler system ^1^ in 1979, Chua model ^2^ in 1986, and a list of about 18 systems not previously known provided by Sprott ^3^ in 1994. For these systems and others, not only chaotic behaviors but also other dynamics, e.g. integrability and global dynamics, were studied. See for instance ^4– 8^.

However, interest in the Lorenz system has not diminished. In 1999, Chen and Ueta proposed a dual system with a chaotic attractor that is not equivalent to the Lorenz attractor ^9^ and in 2002, Lü and Chen proposed a new chaotic system ^10^. A more general Lorenz-type system was defined in 2006 ^11^ and many studies are related to common properties of the systems in this family.

In a class of systems, not only general but also particular properties are important. Some specific systems in the Lorenz family have specific properties and/or applications and deserve to be separately studied. This is the case for the *T*-system introduced in 2008 in 12. This is a 3D autonomous quadratic polynomial system with three real parameters *a*, *b*, *c*, given by

$$\dot{x}=a(y-x),\;\dot{y}=(c-a)x-axz,\;\dot{z}=-bz+xy.\;(1)$$

Properties and potential applications of system ( 1) have been reported in recent years in many papers. For example, the existence of heteroclinic orbits in the *T*-system connecting equilibrium points was studied using Lyapunov functions in 13 or the Shilnikov method in 14. The Hopf bifurcations ^15^ and bifurcations with delayed feedback ^16^ in the *T*-system were studied using analytical methods and a classification of chaotic regimes using competitive modes was realized in 17. Some methods for the control of its chaotic dynamics and synchronization were proposed in 18– 20. General techniques for finite-time stabilization of 3D chaotic systems were exemplified using the *T*-system ^21^. A fractional-order *T*-system was derived and studied in 22. It was shown that system ( 1) has potential applications in secure communications ^23– 25^. These prove the interest in the study of system ( 1).

The aim of the present work is to prove the coexistence of a chaotic attractor together with two isolated unstable periodic orbits in the *T*-system ( 1), to describe properties of global dynamics in the cases when it has *algebraic invariant surfaces*, and to correlate these results with other information obtained using different dynamical methods.

We note that for the coexistence of limit cycles and chaotic attractor, Sprott, Wang and Chen ^26^ provided (perhaps) the first example, namely

$$\dot{x}=yz+a,\;\dot{y}=x^{\text{2}}-y,\;\dot{z}=1-4x,$$

showing the coexistence of a point attractor, a chaotic attractor and a stable limit cycle when the parameter *a* was chosen suitably. Li and Sprott ^27^ constructed an example given by

$$\dot{x}=y+yz,\;\dot{y}=-xz+yz,\;\dot{z}=-ax-xy+b,$$

which has five coexisting attractors: a limit cycle, two equilibria and two strange attractors in different attractor basins for suitably chosen values of the parameters.

We remark that the periodic orbit in 26, 27 is an attractor (a stable limit cycle). In this work, the twin isolated periodic orbits we obtain are unstable and have a one dimensional unstable invariant manifold and a one dimensional stable invariant manifold. Later on, we will show that the *T*-system is a special case of the generalized Lorenz system, which had not exhibited the coexistence of a chaotic attractor with isolated periodic orbits in previous studies.

The paper is structured as follows: *Bifurcations, chaos, unstable periodic orbits* is devoted to the study of the bifurcations of *T*-systems and contains our new discovery, i.e. the coexistence of a chaotic attractor with isolated unstable periodic orbits; in *First integrals and invariant algebraic surfaces* the *T*-systems are identified as having algebraic invariant surfaces; and their global dynamics are analysed in *Global dynamics of the T-system with invariant algebraic surfaces*. Finally, conclusions and discussions are presented.

## Bifurcations, chaos, unstable periodic orbits

If *a* = 0 system ( 1) is linear, it has minor interest. In what follows we will focus on the case *a* > 0 (the case *a* < 0 can be treated similarly).

Using the transformation of variables

$$\;\tilde{x}=\frac{x}{\sqrt{a}},\;\tilde{y}=\frac{y}{\sqrt{a}},\;\tilde{z}=z,\;\tau =at,\;(2)$$

and the transformation of parameters ( *a, b, c*) ↦ ( *a, m, n*) given by $m=\frac{c}{a}-1$ and $n=\frac{b}{a}$ , where *a* > 0, system ( 1) is topologically equivalent to

$$\;\dot{x}=y-x,\;\dot{y}=mx-xz,\;\dot{z}=-nz+xy,\;(3)$$

where "~" was neglected for the sake of simplicity. We notice that the transformation of parameters is invertible. System ( 3) has the advantage of having only two parameters.

### Equilibria and their stability

A stable focus in a 3D system is an equilibrium that has a negative eigenvalue and two complex conjugate eigenvalues with negative real parts, while a saddle focus is an equilibrium that has a negative (or positive) eigenvalue and two complex conjugate eigenvalues with positive (respectively negative) real parts.

**Theorem 1.** *For m* > 0 *and n* > 0, *system* ( 3) *has three equilibria:*

$$O=(0,0,0)\;\text{and}\;P_{\pm }=\left(\pm \sqrt{mn},\pm \sqrt{mn},m\right).$$

*Moreover, the following statements hold*.

*(a)  The equilibrium point O is a saddle with* dim ( *W ^u^* ( *O*)) = 1 *and* dim ( *W ^s^* ( *O*)) = 2. *The equilibria P*_±_ *are stable foci if m*( *n* – 1) + *n* + 1 > 0, *or saddle foci with* dim ( *W ^u^* ( *P*_±_)) = 2 *and* dim ( *W ^s^* ( *P*_±_)) = 1 *if m*( *n* – 1) + *n* + 1 < 0.
*(b)  If* $m=m_{H}\;\text{:=}\;\frac{1+n}{1-n}$ *and* 0 < *n* < 1, *system* ( 3) *undergoes a nondegenerate Hopf bifurcation at P*_±_. *Moreover, the equilibria P*_±_ *are both unstable weak foci on the two dimensional center manifold, and for* 0 < *m _H_* – *m* ≪ 1 *from each of P*_±_ *bifurcates a limit cycle which has a one dimensional unstable manifold and a one dimensional stable manifold*.

*Proof:* the existence of equilibria follow from easy calculations.

*a*). The properties on *O* follows from the observation that the characteristic polynomial of system ( 3) at *O*, i.e.

$$f_{O}(\text{λ})=(\text{λ}+n)({\text{λ}}^{\text{2}}+\text{λ}-m),$$

has one positive and two negative roots.

The properties of *P*_±_ follow from their characteristic polynomials

$$f_{P_{\pm }}(\text{λ})={\text{λ}}^{\text{3}}+(n+1){\text{λ}}^{\text{2}}+(mn+n)\text{λ}+2mn,\;(4)$$

which has a negative root λ _1_ < 0 and two complex conjugated roots λ _2,3_ = α ± i *β*. Here we have used the fact that the equation

$$x^{\text{3}}-px^{\text{2}}-qx-r=0\;\text{with}\;r<0\;\text{and}\;p<0,$$

has three roots with negative real parts if and only if *r* + *pq* > 0, or one negative real root and two roots with positive real parts if and only if *r* + *pq* < 0, respectively.

The real part of λ _2,3_ is negative if and only if –2 *mn* + (– *n* –1) (– *mn* – *n*) > 0. Because *n* > 0 this condition reads *m*( *n* – 1) + *n* + 1 > 0.

The real part of λ _2,3_ is positive if and only if –2 *mn* + (– *n* – 1) (– *mn* – *n*) < 0, so *P*_±_ are saddle foci with dim ( *W ^u^* ( *P*_±_)) = 2 and dim ( *W ^s^* ( *P*_±_)) = 1. This proves statement ( *a*).

( *b*). According to the proof of ( *a*), we treat the eigenvalues of system ( 3) at *P*_±_ as a function of *m*, and take

$${\text{λ}}_{2,3}(m)=\alpha (m)\pm \text{i}\omega (m).$$

Standard computations show that *α* ( *m*) = 0 if and only if $m=m_{H}\;\text{:=}\;\frac{1+n}{1-n}$ > 0. In this case $\omega =\sqrt{\frac{2n}{1-n}}>0$ .

Differentiating the relation *f*_*P*_±__ (λ( *m*)) = 0 with respect to *m*, we obtain

$${\frac{d\alpha (m)}{dm}|}_{m=m_{H}}=\frac{1}{2}\frac{{\left(1-n\right)}^{2}\;n}{(3-n)\;n+1-n^{3}}>0.$$

The first Lyapunov coeffcient ^28^ is

$$l_{1}(m_{H})=\frac{\omega (3+\omega^{2})(6+4\omega^{2}+2\omega^{4}+\omega^{6})}{2(1+\omega^{2})(1+6\omega^{2}+5\omega^{4}+\omega^{6})(4+12\omega^{2}+8\omega^{4}+\omega^{6})}.$$

Notice that it is always positive, *l*_1_ ( *m _H_*) > 0.

The above calculations show that when *m* = *m _H_*, system ( 3) restricted to the two dimensional center manifold at *P _+_* always has the equilibrium *P _+_* as an unstable weak focus. By symmetry, when *m* = *m _H_* system ( 3) restricted to the two dimensional center manifold at *P _–_* always has the equilibrium *P _–_* as an unstable weak focus. Combining the analyses of the equilibria *P*_±_ when *m* ≠ *m _H_*, we get that, for $0<\frac{1+n}{1-n}-m\ll 1,$ system ( 3) has a limit cycle bifurcating from each of *P*_±_. Whereas if we use notations from the Stable Manifold Theorem for periodic orbits ( 29, page 225), each of the limit cycles has a two dimensional unstable manifold and a two dimensional stable manifold, which both contain the limit cycle as a one dimensional submanifold and transversally intersect on it. One may conclude that each of the two limit cycles has a one dimensional unstable manifold and a one dimensional stable manifold. This proves the theorem.

### Dynamical zones in the parametric plane

The regions of the plane ( *n*, *m*) where system ( 3) has different dynamical properties are presented in Figure 2, in which the Hopf bifurcation curve ( *h*) given by

$$m=\frac{1+n}{1-n}\;\text{with}\;\text{0}<n<1,$$

splits the plane ( *n*, *m*) into two zones: *R*_3_ and *R*_1_ ∪ *R*_2_.

**Figure 1.**
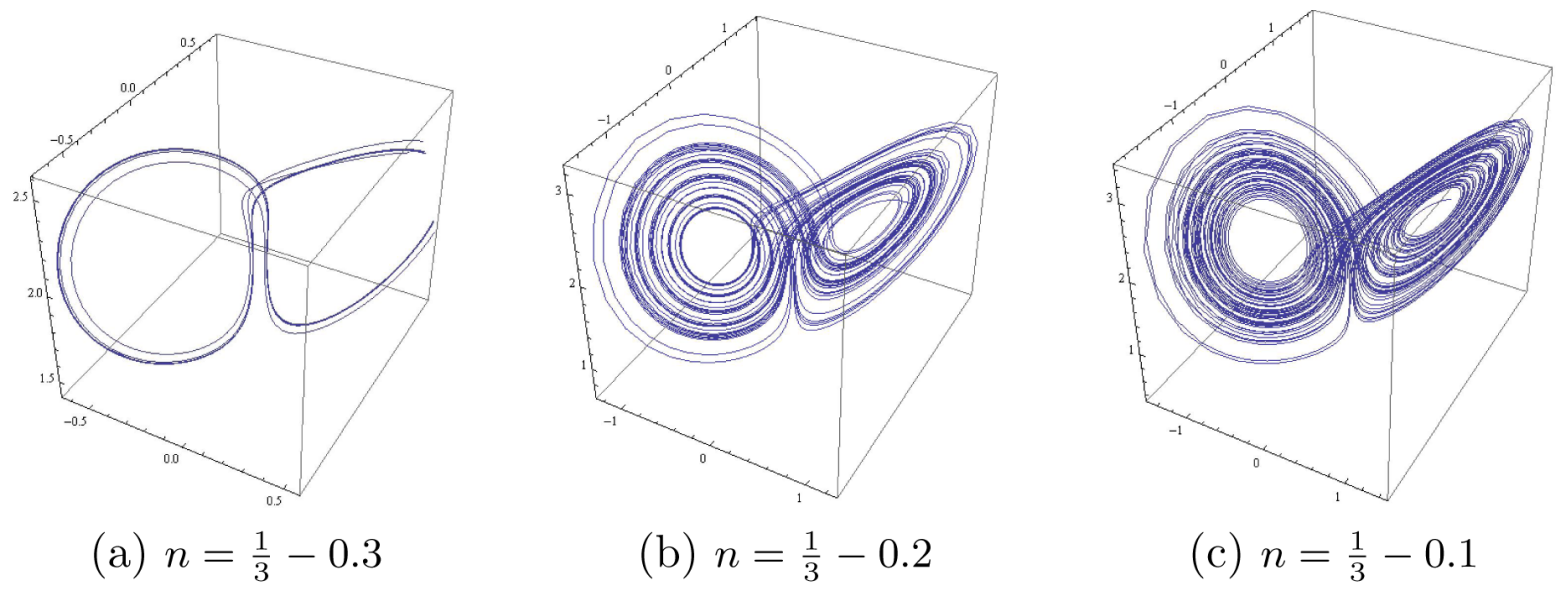
A positive orbit before Hopf bifurcation for *m* = 2 shows the butterfly attractor.

In the region *R*_3_ with $m>\frac{n+1}{1-n}$ , the equilibria *P*_±_ are saddle foci with a one dimensional stable manifold and a two dimensional unstable manifold. The local properties of these equilibria foresee the possibility of existence of the chaotic attractor. Using *m* = 2 and $n=\frac{m-1}{m+1}+K=\frac{1}{3}+K$ for *K* = –0.3, –0.2, –0.1, respectively, and taking the initial values $x(0)=\sqrt{m(m-1)/(m+1)}+0.101,$ $y(0)=\sqrt{m(m-1)/(m+1)}-0.201$ and *z*(0) = *m* + 0.302, we get the three figures showing the positive orbits of the initial value problem, see Figure 1. Note that the initial point of the orbit has a relatively fixed position compared with *P _+_*, and that the positive limit of the orbit becomes much more chaotic when *n* increases from 0 to near the Hopf bifurcation value.

**Figure 2.**
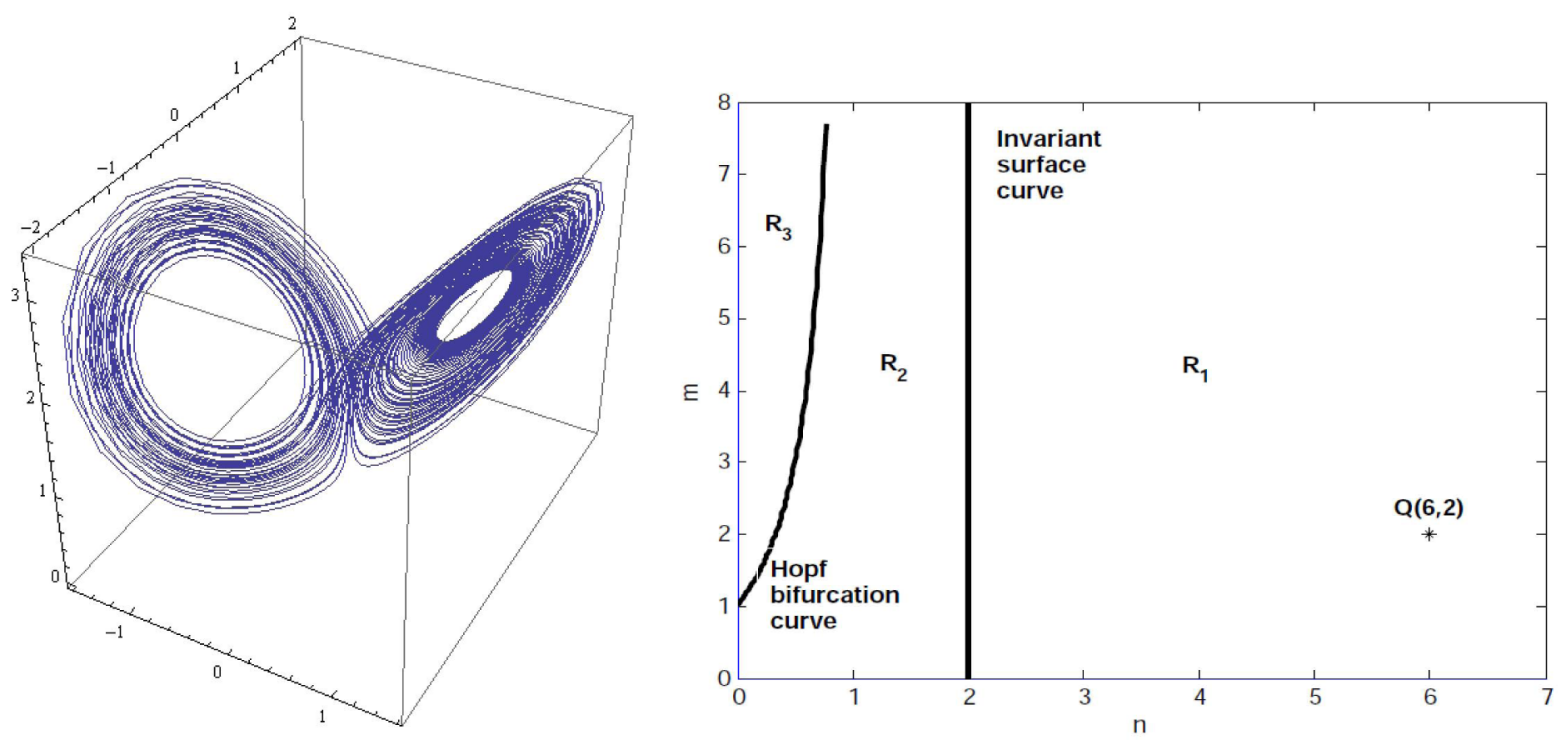
A butterfly attractor exists at the Hopf bifurcation value *m* = 2 and $n=\frac{1}{3}$ (left). Regions with different dynamical properties in the ( *m*, *n*) plane (right).

At the Hopf bifurcation values $n=\frac{m-1}{m+1}$ for *m* > 1, the local structures of the equilibria are similar to the ones for *m* > 1 and $0<n<\frac{m-1}{m+1}$ but now the *P*_±_ are unstable weak foci of order 1 on their center manifolds, respectively, which also forces the existence of chaotic attractor. Figure 2 illustrates such a butterfly attractor for system ( 3) with *m* = 2; where the orbit starts at $x(0)=\sqrt{m(m-1)/(m+1)}+0.101,$ $y(0)=\sqrt{m(m-1)/(m+1)}-0.201$ , *z*(0) = *m* + 0.302.

**Figure 3.**
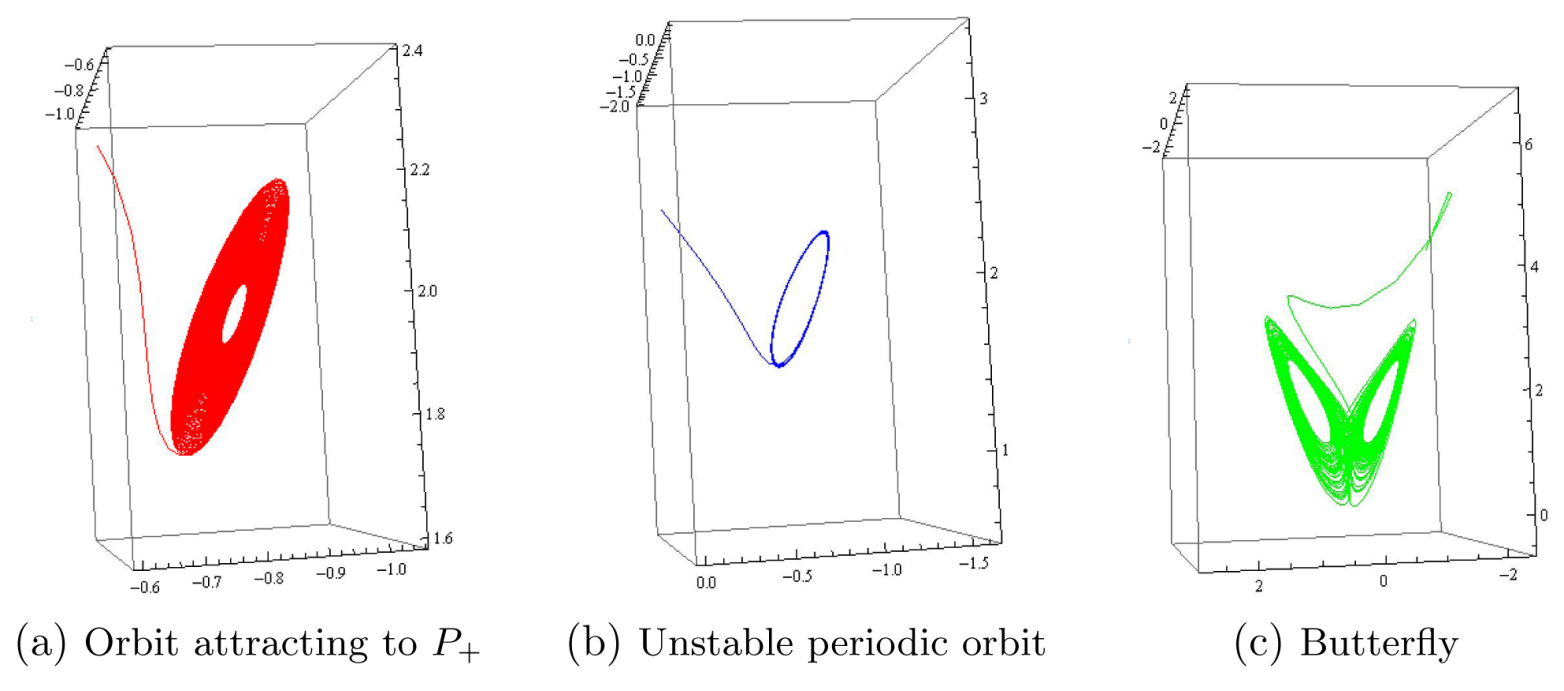
Three orbits of system ( 3) when *m* = 2 and *n* = 1/3 + 0.01, which is after Hopf bifurcation.

When *n* increases from ( *m* – 1)/( *m* + 1) but is less than 2, the parameters are in the region *R*_2_. System ( 3) has the equilibria *P*_±_ as stable foci on the two dimensional stable manifolds $M_{\pm }^{s}$ approaching, respectively, the center manifolds $M_{\pm }^{c}$ of system ( 3) at *P*_±_ when *n* = ( *m* – 1)/( *m* + 1). Then there is a limit cycle bifurcating from each of *P*_±_ on $M_{\pm }^{s}$ , respectively. By the continuity of orbits with respect to parameters we have shown that for 0 < *n* – ( *m* – 1)/( *m* + 1) ≪ 1 system ( 3) has also a butterfly attractor and two additional isolated periodic orbits with a one dimensional unstable manifold and a one dimensional stable manifold. Figure 3 illustrates the stable focus, the periodic orbit and the butterfly attractor of system ( 3) when *m* = 2 and $n=\frac{1}{3}$ + 0.01, where the initial values of the three orbits have the same *x* and *y* coordinates, *x*(0) = *y*(0) = 5.001, but different *z* coordinates, which are *z*(0) = –2.515 in( *a*), –1.424 in ( *b*) and –0.515 in ( *c*), respectively.

**Figure 4.**
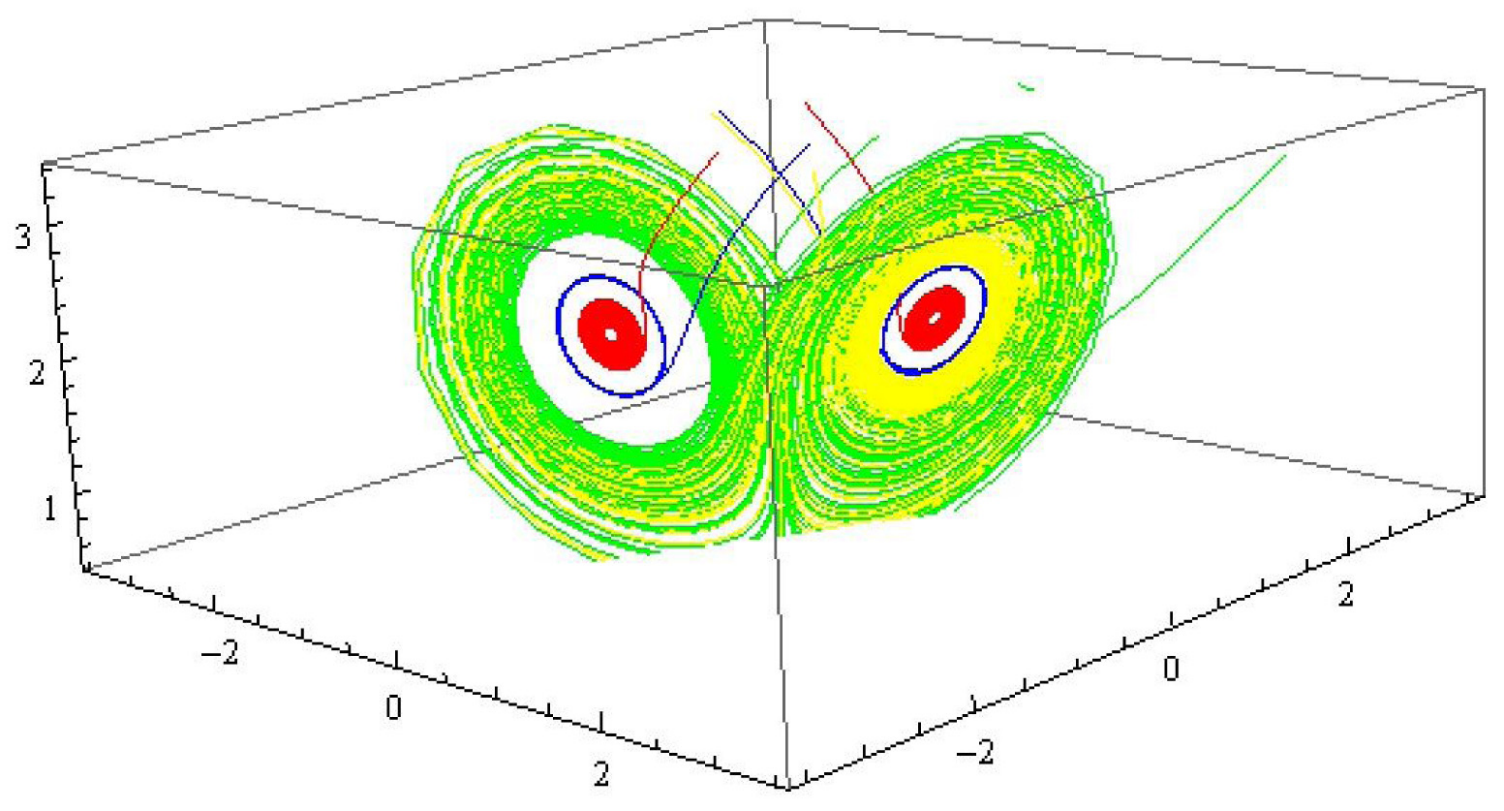
The six positive orbits of system ( 3) when *m* = 2, $n=\frac{1}{3}$ + 0:01: the red ones have limits at *P*_±_, respectively, the blue ones have their limits as one of the two unstable limit cycles on the two dimensional invariant manifolds, and the green and yellow ones approach the butterfly attractor.

Now for system ( 3) with *m* = 2 and $n=\frac{1}{3}$ + 0.01, we take another three initial points, whose *x* and *y* coordinates are the same with *x*(0) = *y*(0) = –5.001, and *z* coordinates are the same as the previous three, i.e. *z*(0) = –2.515, –1.424 and –0.515, respectively. Putting all these six orbits in a unique picture, one gets the figure given in Figure 4, which contains two stable foci, two isolated unstable periodic orbits with, respectively, a one dimensional stable invariant manifold and a butterfly attractor.

In order to get more evidence on the existence of a strange attractor at the Hopf bifurcation values, or after and before these values, we computed the Lyapunov exponents using the method presented in 30 for values of *m* ∈ [1, 4].

**Figure 5.**
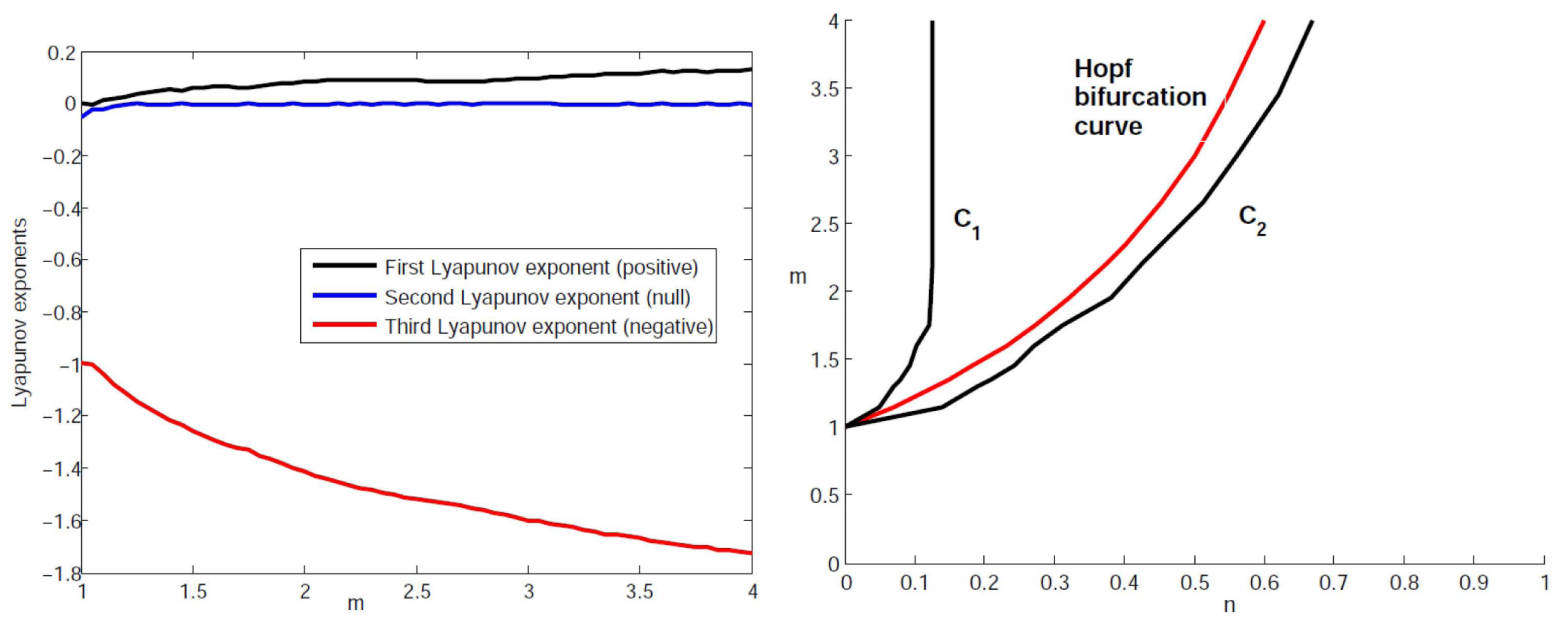
The Lyapunov exponents at the Hopf bifurcation values for *m* ∈ [1, 4] (left). The zone situated between *C*_1_ and *C*_2_ is formed by couples ( *n*, *m*) for which the corresponding system has a strange attractor (right).

By our calculation at the Hopf bifurcation values, it was observed that the first Lyapunov exponent is positive for all values of *m* > 1. Moreover, the first Lyapunov exponent increases when the values of *m* increase. The second Lyapunov exponent is null and the third one is negative, which shows that the butterfly attractor strange and the dynamics of the system in the basin of attraction of this attractor are chaotic ^30^. Figure 5 presents the values of Lyapunov exponents, which were computed for various values of *m*; using a long orbit of ( *x*(0), *y*(0), *z*(0)) = (5.001, 5.001, –2.515).

We note that the existence of a strange attractor before and after the Hopf bifurcation can be also proved through the computation of the Lyapunov exponents for systems whose parameters ( *n*, *m*) are situated near the Hopf bifurcation curve. In Figure 5, the zone situated between the curves *C*_1_ and *C*_2_ is formed by parameters ( *n*, *m*) for which the dynamical system has a positive, a null and a negative Lyapunov exponent, i.e. there is a strange attractor coexisting with two saddle foci *P*_±_ in the region *R*_3_ (i.e. $m>\frac{1+n}{1-n}\text{>}0$ ) or with the two stable equilibria *P*_±_ and the unstable limit cycles in *R*_2_ (i.e. $\text{0}<\frac{1+n}{1-n}-m<\rho ,$ with *ρ* a suitable positive small number).

## First integrals and invariant algebraic surfaces

The purpose of many studies is to understand the global dynamics of systems from the Lorenz family. A first step for this is to study subclasses of these systems, for example, the subclass of systems with invariant algebraic surfaces. Such surfaces in the Lorenz system were studied in 31. Darboux polynomials in Chen and Lü systems were studied in 32– 34 and a generalization is achieved in 35. The non-algebraic integrability of the Chen and Lü systems was studied in 36 and 37, respectively.

The global dynamics of the Lorenz system with invariant algebraic surfaces was studied in 38 and similar studies for the Chen system were performed in 32. The description of the dynamics in generalized Lorenz systems with invariant algebraic surfaces was performed in 39. In this work, our study on the existence of invariant algebraic surfaces of the *T*-system and its related global dynamics will be helpful to understand the disappearance of the chaotic attractor.

Invariant surfaces and first integrals are important tools for understanding the behavior of dynamical systems. A Darboux polynomial of the 3D polynomial differential system

$$\;\dot{x}=P\text{(}x,y,z\text{),}\;\dot{y}=Q\text{(}x,y,z\text{),}\;\dot{z}=R\text{(}x,y,z\text{),}\;(5)$$

with *P*, *Q*, *R* ∈ ℝ[ *x*, *y*, *z*] as the ring of polynomials, is a polynomial *F* ∈ ℂ[ *x*, *y*, *z*] which fulfills

$$\frac{\partial F}{\partial x}P+\frac{\partial F}{\partial y}Q+\frac{\partial F}{\partial z}R=kF,$$

for some polynomial *k* ∈ ℂ[ *x, y, z*], which is called the cofactor ^35^.

If *k* ≠ 0, then *F* = *F*( *x, y, z*) is called a proper Darboux polynomial. If *k* = 0, then *F* = *F* ( *x, y, z*) is a first integral of ( 5), that is $\frac{dF}{dt}|$ _( 5)_ = 0. If system ( 5) has two functionally independent first integrals *F*_1_ ( *x, y, z*) and *F*_2_ ( *x, y, z*) defined in a domain Ω ⊂ ℝ ^3^, then the orbits of the system in Ω are contained in the curves { *F*_1_ ( *x, y, z*) = *h*_1_} ∩ { *F*_2_ ( *x, y, z*) = *h*_2_} for some *h*_1_, *h*_2_ ∈ ℝ, and the system is completely integrable.

If *F* ( *x, y, z*) is a Darboux polynomial of ( 5), then the set

$$S_{F}=\left\{(x,y,z)\in {\mathbb{R}}^{3}\;|\;F(x,y,z)=0\right\}$$

is invariant under the flow of the system ( 5). It justifies its name *invariant algebraic surface.*

A generalized Lorenz system given by

$$\dot{x}=A(y-x),\;\dot{y}=Bx+Cy-xz,\;\dot{z}=Dz+xy,\;(6)$$

was studied in 35, where the existence of invariant algebraic surfaces have been obtained. It has been shown in Theorem 1.1 from 35 that there are only six cases in which system ( 6) has Darboux polynomials, namely: a) *D* = −2 *A*, when the cofactor is *k* = −2 *A*, b) *D* = *C* and *B* = 0, when the cofactor is *k* = 2 *C*, c) *A* = *−C* and *D* = *C,* when the cofactor is *k* = 2 *C*, d) *A* = − *C*/3 and *D* = 0, when the cofactor is *k* = 4 *C*/3, e) *A* = − *C* and *D* = 4 *C*, when the cofactor is *k* = 4 *C*, f) *B* = 2 *A* + *C* and *D* = −6 *A* − 2 *C*, when the cofactor is *k* = −4 *A*. In 40 the authors found the seventh case, *C* = *D* = 0, when system ( 6) has the first integral *F*( *x , y, z*) = *y* ^2 ^ + *z* ^2 ^ − 2 *Bz*. The global dynamics in each case were described in 40 and 39. We will apply the results of these papers to study system ( 3).

**Theorem 2**. *System ( 3) with a > 0 has algebraic invariant surfaces if and only if*

( *a*) *either n* = 2 *, when the algebraic invariant surface is x* ^2^ − 2 *z* = 0,
  ( *b*) *or m* = 2, *n* = 6 *when the algebraic invariant surface is*
$$x^{4}-4x^{2}z-4y^{2}+16xy-16x^{2}=0,$$
  ( *c*) *or n* = 0, *when there are infinitely many algebraic invariant surfaces with the equation*
$$y^{2}+z^{2}-2mz=cst.$$

*Proof:* The proof is a consequence of Theorem 1.1 in 35, which shows that system ( 6) admits proper Darboux polynomials only in two cases, namely *D* = –2 *A* (when the Darboux polynomial is ${\bar{F}}_{1}$ ( *x*, *y*, *z*) = *x* ^2^ – 2 *Az*), and *B* = 2 *A* and *D* = –6 *A* (when the Darboux polynomial is ${\bar{F}}_{2}$ ( *x*, *y*, *z*) = *x* ^4^ – 4 *Ax* ^2^ *z* – 4 *A* ^2^ *y* ^2^ + 16 *A* ^2^ *xy* – 16 *A* ^2^ *x* ^2^). These conditions read *n* = 2, *m* = 2 and *n* = 6 in system, respectively ( 3). The corresponding invariant surfaces, obtained from the Darboux polynomials through the transformation ( 2) are *x* ^2^ – 2 *z* = 0 and *x* ^4^ – 4 *x* ^2^ *z* – 4 *y* ^2^ + 16 *xy* – 16 *x* ^2^ = 0, respectively.

In the case *n* = 0, we have *C* = *D* = 0 so the polynomial

$$\bar{F}\;(x,y,z)=y^{2}+y^{2}-2mz$$

is a first integral of system ( 6). From ( 2) it results that *F* ( *x*, *y*, *z*) = *y* ^2^ + *z* ^2^ – 2 *mz* is a first integral of ( 3). In this case we have infinitely many invariant surfaces with the equation *F* ( *x*, *y*, *z*) = *cst*. Consequently, ℝ ^3^ is practically foliated by the invariant cylinders *y* ^2^ + ( *z* – *m*) ^2^ = *r* ^2^ for 0 < *r* ∈ ℝ.

## Global dynamics of the T-system with invariant algebraic surfaces

In the following we will denote the orbit of system ( 3) passing *q*_0_ ∈ ℝ ^3^ by *O* ( *q*_0_) = { *φ _t_* ( *q*_0_) | *t* ∈ ℝ} and by α ( *q*_0_) ( *ω* ( *q*_0_)), the α ( *ω*) limit sets of *O* ( *q*_0_).

Theorem 2 in 39 shows that, for *a* ≠ 0, all orbits of the generalized Lorenz system ( 6) starting from outside the algebraic invariant surface *S _F_* are heteroclinic: they either all positively approach the surface and negatively go to infinity (i.e. *ω* ( *q*_0_) ⊂ *S _F_* and α ( *q*_0_) = ∞), or converse.

We now present the global dynamics of the *T*-systems, and its sketch proof for completeness. Set

$$\begin{array}{l} S_{F_{1}}\;:\;x^{2}-2z=0,\\ S_{F_{2}}\;:\;x^{2}-4x^{2}z-4y^{2}+16xy-16x^{2}=0,\\ \;S_{r}\;:\;y^{2}+(z-m{)}^{2}=r^{2},\;0<r\in \mathbb{R}. \end{array}$$

**Theorem 3.** *Let a* > 0, *and consider the T–system having an invariant algebraic surface. The following statements hold.*

*(a) n* = 2. *S*_*F*_1__ *is an invariant algebraic surface.*
  ( *a*_1_) *For m* < 0 *system* ( 3) *has only an equilibrium O* = (0, 0, 0), *which is situated on S*_*F*_1__, *and is globally asymptotically stable in* ℝ ^3^.
  ( *a*_2_) *For m* > 0 *system* ( 3) *has three equilibria: O and P*_±_ = (± *x*_0_, ± *x*_0_, *m*), *x*_0_ = $\sqrt{2m}$ , *which are situated on S*_*F*_1__. *The equilibria P _+_ and P _–_ are asymptotically stable and the equilibrium O is a saddle with a two dimensional stable manifold W ^s^* ( *O*) *and a one dimensional unstable manifold W ^u^* ( *O*). *The two branches of W ^u^* ( *O*) *are included in S*_*F*_1__, *and heteroclinic to P*_+_ *and P*_–_, *respectively.*
  ( *a*_3_) *All orbits with initial points q*_0_ ∈ ℝ ^3^ *not on the the invariant algebraic surface are heteroclinic:* ω ( *q*_0_) ⊂ *S*_*F*_1__ *and α* ( *q*_0_) = ∞. *If m* < 0 *then* ω ( *q*_0_) = *O*. *If m* > 0 *then* ω ( *q*_0_) = *O* *or* ω ( *q*_0_) = *P*_+_ *or* ω ( *q*_0_) = *P*_–_.
( *b*) *m* = 2 *and n* = 6. *S*_*F*_2__ *is an invariant algebraic surface*.
  ( *b*_1_) *The equilibria of system* ( 3) *are O* = (0, 0, 0) *and P*± = ±2 $\sqrt{12}$ , ±2 $\sqrt{12}$ , 2), *which are situated on *S*_*F*_2__. They have the same properties as those in (a _2_) with *S*_*F*_2__ replacing SF1*.
  ( *b*2) *All orbits with initial points* *q*_0_ ∈ ℝ ^3^ *not on* *S*_*F*_2__ *are heteroclinic: α* ( *q*_0_) = ∞ *and either* ω ( *q*_0_) = *O or* ω ( *q*_0_) = *P*_+_ *or* ω ( *q*_0_) = *P*_–_.
( *c*) *n* = 0. *S _r_ for all r* > 0 *are invariant*.
  ( *c*_1_) *Each S _r_ contains two equilibria of* ( 3): *P*± = (0, 0, *m* ± *r*), *in which P*_+_ *is a saddle and P*_–_ *is asymptotically stable*.
  ( *c*_2_) *All orbits on S _r_ converge to P*_–_, *except the orbits starting from the stable manifold of P*_+_.

*Proof:* ( *a*). That *S*_1_ is invariant follows from Theorem 2.

( *a*_1_) and ( *a*_2_). The results concerning equilibria and their stability are obtained from typical calculations.

We show that *W ^u^* ( *O*) ⊂ *S*_*F*_1__. In fact, consider the Lyapunov function *V* ( *x*, *y*, *z*) = ( *x* ^2^ – 2 *z*) ^2^. It is a decreasing function along the trajectories because

$$\frac{dV}{dt}(\varphi_{t}(x,y,z))=-4{\left(x^{2}-2z\right)}^{2}\leq 0.$$

We observe also that *V* ( *x*, *y*, *z*) = 0 if and only if ( *x*, *y*, *z*) ∈ *S*_*F*_1__.

The above proofs show that all orbits positively attract to the surface *S*_1_. System ( 3) restricted to *S*_*F*_1__ becomes

$$\dot{x}=(y-x)\;\text{=}:\;R(x,y),\;\dot{y}=mx-\frac{x^{3}}{2}\;\text{=}:\;S(x,y).\;(7)$$

Since $\frac{\partial R}{\partial x}+\frac{\partial S}{\partial y}=-1<0$ , it follows from the Bendixon theorem ^41^ that system ( 7) has no periodic orbits. Then the conclusions in ( *a*_1_) and ( *a*_2_) follow easily.

( *a*_3_). Let *q*_0_ ∈ ℝ ^3^\ *S*_*F*_1__, *r* ∈ α ( *q*_0_) and *s* ∈ ω ( *q*_0_). For the function *V* defined above, one has

$$V(r)>V(q_{0})>V(s)\geq 0$$

it results that *r* ∉ *S*_*F*_1__ and *s* ∈ *SF*_1_ , i.e. α ( *q*_0_) = ∞ and *ω* ( *q*_0_) ⊂ *S*_*F*_1__. Since system ( 7) has no limit cycles, *ω* ( *q*_0_) is an equilibrium.

If *q*_0_ ∈ *W ^s^* ( *O*) then *ω* ( *q*_0_) = *O*. If *q*_0_ ∈ ℝ ^3^\ *W ^s^* ( *O*) then *ω* ( *q*_0_) = *P*_+_ or *ω* ( *q*_0_) = *P*_–_. This proves statement ( *a*).

( *b*). That *S*_*F*_2__ is invariant follows from Theorem 2.

The other substatements can be proved by similar arguments as those in statement ( *a*), instead using the Lyapunov function

$$V(x,y,z)={\left(x^{4}-4x^{2}z-4^{2}y^{2}+16xy-16x^{2}\right)}^{2},$$

and the fact

$$\frac{dV}{dt}(\varphi_{t}(x,y,z))=-8V\;(x,y,z)\leq 0,$$

which ends the proof of statement ( *b*).

( *c*). That *S _r_* 's are invariant follows from Theorem 2, because *y* ^2^ + *z* ^2^ –2 *mz* is a first integral of system ( 3).

( *c*_1_). System ( 3) has infinitely many equilibria *E* = (0, 0, *z*) , *z* ∈ ℝ. When restricted to the cylinder *S _r_* it has two equilibria: *P*_+_ = (0, 0, *m* + *r*) and *P*_–_ = (0, 0, *m* – *r*), which have the eigenvalues ${\text{λ}}_{1,2}=-\frac{1}{2}\pm \frac{\sqrt{1+4r}}{2}$ and ${\text{λ}}_{3,4}=-\frac{1}{2}\pm \frac{\sqrt{1-4r}}{2}$ , respectively. These expressions of the eigenvalues show that *P*_+_ is a saddle, and that *P*_–_ is a stable node if *r* ≤ 1/4 or a stable focus if *r* > 1/4. Because of invariance of *S _r_*, it results that the stable and the unstable manifolds of *P*_+_ are contained in *S _r_*.

( *c*_2_). On the finite part of the cylinder there are not periodic orbits, because the divergence of the system on *S _r_* is negative, and the infinity of the cylinder is the endpoint of the *x*–axis. From the analysis on Poincaré compactification we know that the infinity at the *x*–direction is unstable. These results show that all orbits converge to *P*_–_, except the ones starting from the stable manifold of *P*_+_. This proves the statement. The proof of the theorem is completed.

In the zone *R*_1_ ∪ *R*_2_, the equilibria *P*_+_, *P*_–_ are stable foci and *O* is saddle with dim ( *W ^u^* ( *O*)) = 1 and dim ( *W ^s^* ( *O*)) = 2.

Zone *R*_1_, i.e. *n* ≥ 2 all positive orbits of ( 3) are attracted by one of the equilibrium points *O*, *P*_+_, *P*_–_ and there are two heteroclinic orbits connecting *O* with *P*_+_, and *O* with *P*_–_. These heteroclinic orbits form the unstable manifolds of *O*. Associated to system ( 3), the proof can be shown by using the Lyapunov function

$$V=2n(n-2){\left(y-x\right)}^{2}+2{\left(x^{2}-nz\right)}^{2}+(n-2){\left(x^{2}-mn\right)}^{2},$$

and its derivative along system ( 3)

$${\frac{dV}{dt}|}_{\left(3\right)}=-4n{\left(x^{2}-nz\right)}^{2}-4n(n-2){\left(y-x\right)}^{2}.$$

The limit between zones *R*_1_ and *R*_2_ is the line *n* = 2. If *n* = 2, system ( 3) has the algebraic invariant surface *S*_*F*_1__. At *n* = 2 the dynamics of the system are qualitatively the same as what happens at ( *n*, *m*) ∈ *R*_1_. In the zone *R*_1_ one can find the point *Q* = ( *n*, *m*) = (6, 2). The invariant surface of ( 3) is *S*_*F*_2__. The dynamics of ( 3) corresponding to ( *n*, *m*) = (6, 2) are not qualitatively diffierent from those of systems obtained from other values of the parameters in *R*_1_, the only specific property is that the orbits starting from the invariant surface *S*_*F*_2__ (in particular the heteroclinic connections) do not leave *S*_*F*_2__.

## Conclusions and discussions

We obtained in this work new properties of the *T*-system. Near the Hopf bifurcation curve $m=\frac{n+1}{1-n}$ with 0 < n < 1, the *T*-system could have a butterfly attractor, and after Hopf bifurcation the system exhibits the coexistence of a butterfly attractor and two isolated unstable periodic orbits with a one dimensional stable manifold, respectively. When *n* = 0 or *n* = 2 the chaotic attractor disappears, because of the existence of the invariant algebraic surface of system ( 3). Each orbit converges in either positive time or negative time to an equilibrium on the invariant surface.

The complex dynamics of the *T*-system is diffierent from the dynamics of Lorenz, Chen and Lü systems, in which the Hopf bifurcation is the first step toward a complex (chaotic) behavior. Here, the chaotic behavior for the *T*-systems happens both before and after Hopf bifurcation.

## Data availability

All data underlying the results are available as part of the article and no additional source data are required.
