# Supplementary material for: Coexistence of chaotic attractor and unstable limit cycles in a 3D dynamical system
Source: Open Res Eur. 2021 May 17;1:50. [Version 1] doi: 10.12688/openreseurope.13590.1 (PMC10446012; doi:10.12688/openreseurope.13590.1)
Supplement: Peer review history

# Peer review: Reviewer response for version 1

**DOI:** 10.21956/openreseurope.14660.r27620

**Published:** 2021-10-06

**Authors:** Regilene Oliveira

**Affiliations:** 1 Instituto de Ciências Matemáticas e de Computação, Universidade de São Paulo, São Paulo, Brazil

Since the first chaotic model was presented, i.e., the Lorenz system, there is a huge number of papers devoted to the studies of such class of systems. Among the chaotic systems that are closely related but not topologically equivalent to the Lorenz system, it is worth mentioning the Chen system and the Lü and Chen systems. Sprott embarked upon an extensive search for autonomous three-dimensional chaotic systems by computer simulation. Research about chaos have concentrated on not only finding new and interesting chaotic systems or studying chaos control and chaos synchronization but also analyzing their local and global characteristics which are essential for what is meant by chaos. There have been some detailed bifurcation analyses about the obtained chaotic systems. Researches show that chaotic systems have rich nonlinear dynamics and they are of significance in practical applications.

 This papers follows such idea. In this paper the authors investigate the global dynamics of the T-systems. Such system were introduced by the second author of this paper of 2008 and it is a special case of a generalized Lorenz system. Recently, properties and potential applications of T-systems have been reported, but in this paper it is a first time that the coexistence of limit cycles and chaotic attractors were described for such system.

 The main result of this paper guarantee that the 2-parametric system known as T-systems admits a twin isolated periodic orbits bifurcating from two unstable weak focus together with a strange attractor for specific values of the parameters. More specifically, the author show that there exist a strange attractor before and after the Hopf bifurcation of the T-system whose parameters (n,m) are situated near of the Hopf bifurcation curve m=(1+n)/(1-n), for 0 < n< 1. This situation shows that the dynamics of the T-systems are different the dynamics of Lorenz, Chen and Lu systems.

 The coexistence of periodic, chaotic, and stable fixed points discovered in this work has not yet been reported for a T-system and thus merits dissemination.

Is the study design appropriate and does the work have academic merit?

Yes

Is the work clearly and accurately presented and does it cite the current literature?

Yes

If applicable, is the statistical analysis and its interpretation appropriate?

Not applicable

Are all the source data underlying the results available to ensure full reproducibility?

No source data required

Are the conclusions drawn adequately supported by the results?

Yes

Are sufficient details of methods and analysis provided to allow replication by others?

Yes

Reviewer Expertise:

Qualitative theory of ordinary differential equations

I confirm that I have read this submission and believe that I have an appropriate level of expertise to confirm that it is of an acceptable scientific standard.

# Peer review: Reviewer response for version 1

**DOI:** 10.21956/openreseurope.14660.r26918

**Published:** 2021-06-01

**Authors:** Valery Romanovski

**Affiliations:** 1 Center for Applied Mathematics and Theoretical Physics, University of Maribor, Maribor, Slovenia

The authors consider the problem of coexisting of a limit cycle and a chaotic attractor in three dimensional systems of ODEs. Examples of systems having a stable limit cycle and a chaotic attractor were known earlier. In this paper systems with two unstable limit cycles and a chaotic attractor are found. To achieve this goal the authors studied in details a family of the so-called T-systems introduced by the second author in 2008. The considered family is a three dimensional quadratic system depending on three parameters, which can be reduced after a change of variables to a system depending on two positive parameters.

 The authors first study singular points of the system showing that it can have the saddle and focus type equilibriums and identifying the corresponding values of parameters. Then it is proved that an unstable limit cycle bifurcates from each of two symmetric foci as the result of a Hopf bifurcation. Performing the investigation of global dynamical properties of the system the authors show that a butterfly type attractor exists in the system for values of parameters in a neighborhood of the Hopf bifurcation point. Thus, the dynamical behavior of the T-system is different from the dynamics of Lorenz, Chen and Lü systems, since in the investigated system the chaotic attractor exists both before and after the Hopf bifurcation, whereas in the systems mentioned above the Hopf bifurcation can be considered as the first step toward the chaotic behavior.

 For the studied systems the necessary and sufficient conditions for existence of algebraic invariant surfaces are obtained and the dynamics of systems with such surfaces is investigated. In particular it is shown that the chaotic attractor disappears when an invariant surface appears in the system.

 The obtained theoretical results are confirmed by numerical simulations.

 Overall, the article presents an interesting and original study and, in my opinion, it merits indexing at Open Research Europe.

Is the study design appropriate and does the work have academic merit?

Yes

Is the work clearly and accurately presented and does it cite the current literature?

Yes

If applicable, is the statistical analysis and its interpretation appropriate?

Not applicable

Are all the source data underlying the results available to ensure full reproducibility?

No source data required

Are the conclusions drawn adequately supported by the results?

Yes

Are sufficient details of methods and analysis provided to allow replication by others?

Yes

Reviewer Expertise:

Ordinary differential equations.

I confirm that I have read this submission and believe that I have an appropriate level of expertise to confirm that it is of an acceptable scientific standard.
